# Detection of Extracellular Traps in Canine Steroid-Responsive Meningitis-Arteritis

**DOI:** 10.3389/fvets.2022.863579

**Published:** 2022-05-03

**Authors:** Jan C. Wohlsein, Marita Meurer, Jasmin Neßler, Peter Wohlsein, Maren von Köckritz-Blickwede, Wolfgang Baumgärtner, Andrea Tipold

**Affiliations:** ^1^Department of Small Animal Medicine and Surgery, University of Veterinary Medicine Hannover, Foundation, Hannover, Germany; ^2^Department of Biochemistry, University of Veterinary Medicine Hannover, Foundation, Hannover, Germany; ^3^Research Center of Emerging Diseases and Zoonosis, University of Veterinary Medicine Hannover, Foundation, Hannover, Germany; ^4^Department of Pathology, University of Veterinary Medicine Hannover, Foundation, Hannover, Germany

**Keywords:** extracellular traps (ETs), steroid-responsive meningitis-arteritis (SRMA), vasculitis, meningitis, immunofluorescence microscopy, non-infectious, citrullinated histone H3 (H3Cit)

## Abstract

Extracellular traps (ETs) are DNA networks formed by immune cells to fight infectious diseases by catching and attacking pathogenic microorganisms. Uncontrolled ET formation or impaired ET clearance can cause tissue and organ damage. Steroid-responsive meningitis-arteritis (SRMA) represents an immune-mediated, presumably non-infectious, purulent leptomeningitis and fibrinoid-necrotizing arteritis and periarteritis of young-adult dogs. Chronic and recurrent cases of SRMA are characterized by lymphohistiocytic inflammatory cell infiltration in the meninges and perivascular tissue. This study aimed to identify extracellular traps in dogs with SRMA, a model for immune-mediated diseases in the central nervous system (CNS). Hematoxylin and eosin-stained samples of two young dogs with chronic, recurrent SRMA were examined by light microscopy for characteristic lesions and consecutive slices of affected tissues were stained for detection of ETs by immunofluorescence microscopy using antibodies against DNA–histone-1 complexes, myeloperoxidase, and citrullinated histone H3. Histology revealed purulent and lymphohistiocytic leptomeningitis (*n* = 2/2) with meningeal periarteritis (*n* = 2/2) and periadrenal located lymphohistiocytic periarteritis (*n* = 1). Extracellular DNA networks and inflammatory cell infiltrates of macrophages, neutrophil granulocytes, and lymphocytes were detected in the subarachnoid space of the leptomeninx (*n* = 2/2) and perivascularly in meningeal (*n* = 2/2) as well as periadrenal vessels (*n* = 1/1). In summary, extracellular DNA fibers and attached ET markers are detectable in affected perivascular and meningeal tissues of dogs suffering from SRMA. The proof of principle could be confirmed that ETs are present in canine, inflammatory, and non-infectious CNS diseases and possibly play a role in the pathogenesis of SRMA.

## Introduction

Several immune cells of the innate immune system, including neutrophils, eosinophils, monocytes, mast cells, and basophils, are capable of producing extracellular DNA traps (ETs) (1–5).

Extracellular trap formation can be differentiated in three pathways: suicidal, vital, and mitochondrial mechanisms (1, 2, 6–11). The term ETosis was created to define this distinct cell death apart from necrosis and apoptosis describing the suicidal pathway of ET formation (6, 12). This particular cell death involves the resolution of nuclear membrane, decondensation of chromatin, and mixing with granule components followed by release of ETs after permeabilization of the cell membrane (1, 6). ET-related proteins and components such as myeloperoxidase (MPO), citrullinated histone H3 (H3Cit), and DNA–histone complexes were used with antibody-based techniques to co-stain these specific ET markers (13).

The view on ETosis and suicidal ET formation had to be renewed after a groundbreaking discovery in 2012. Pilsczek et al. (7) described viable neutrophils performing phagocytosis and migration after the release of ETs during an acute infection with *Staphylococcus aureus*. The term ET formation was expanded and divided up in suicidal and vital way of ET release (7, 10, 11). Suicidal ET formation is reactive oxygen dependent and pursues after 3–8 h, whereas vital ET formation is reactive oxygen independent and performed in 5–60 min (6, 7, 14). The third way of ET formation due to mitochondrial DNA release by viable cells is not entirely understood (9). In this study, the term ET formation is used to resume all the different ways of creating extracellular DNA traps regardless of the cell origin and type of ET metabolism.

These extracellular DNA formations are composed of a scaffold of decondensed chromatin fibers equipped with granule proteins [e.g., myeloperoxidase (MPO) and neutrophil elastase (NE)], nuclear proteins [e.g., citrullinated histone H3 (H3Cit)], and antimicrobial enzymes forming web-like structures (1, 13, 15, 16). Beyond phagocytosis, degranulation, and creation of reactive oxygen species, ET formation is a genuine extracellular strategy especially of neutrophils to kill, disarm, and entrap invading pathogens (1, 17, 18).

Recently, study shifted from infectious to non-infectious diseases investigating potential impact and therapeutic opportunities of extracellular trap release and degradation. On one hand, ET formation is another effective antimicrobial mechanism of the innate immune system combating different pathogens; on the other hand, excessive ET expression, unregulated ET release, and insufficient ET clearance can cause detrimental effects and lead to or are associated with ET-related pathologies [“ETopathies” (19, 20)]: endothelial or epithelial tissue damage (21–23), pancreatitis (24, 25), autoimmune diseases (26, 27), thrombosis (16), vasculitis (28), and cancer (29, 30).

In dogs, neutrophil extracellular traps (NETs) were recently described in infectious diseases such as parasitic infections with *Toxoplasma gondii, Trypanosoma cruzi*, and *Dirofilaria immitis* (31–33) and bacterial infections such as pyometra caused by *Escherichia coli* and *Streptococcus species* (34) and NETs were isolated from pleural and abdominal effusions in septic dogs (35). However, the influence on the pathogenesis and prognosis of ET formation in canine non-infectious diseases, especially in the CNS, still has to be elucidated. NETs have an impact on the immune system in canine immune-mediated hemolytic anemia (36–38), on clot formation and canine immunothrombosis (16, 39).

Steroid-responsive meningitis-arteritis (SRMA) is an immune-mediated, systemic, inflammatory, and presumably non-infectious disorder predominantly in young-adult and medium-to-large-sized dogs (40). The disease affects typically 6 to 18 month-old dogs with a possible range of 3 months to 9 years (40–42). SRMA can occur in any dog breed, but is overrepresented in Bernese mountain dogs, Boxers, Beagles (43, 44), Nova Scotia Duck Tolling Retrievers, Weimaraners, and Petit Basset Griffon Vendéens (45). A German study showed a sex predisposition for male individuals (46), but other studies do not show significant difference in sex distribution of this disease (47, 48). The assembly of signalment, clinical signs, and laboratory findings of CSF and blood analysis associated with a quick clinical improvement after application of immunosuppressive therapy with glucocorticosteroids and an exclusion of an infectious etiology lead to the antemortem diagnosis of SRMA (40, 48–50).

The typical, acute form of SRMA is characterized by recurrent fever, cervical hyperesthesia, neck rigidity, stiff gait, reluctance to move, and depression. Laboratory findings of the acute form include a moderate-to-severe, non-degenerative neutrophilic pleocytosis of the cerebrospinal fluid (CSF) and blood profiles show a neutrophilic leukocytosis with left shift (44, 51). Furthermore, elevated immunoglobulin A levels in serum and CSF serve as diagnostic tool (44, 52). Levels of acute phase proteins such as C-reactive protein, serum amyloid A, haptoglobin (53), or neutrophil gelatinase-associated lipocalin (54) are elevated in the acute disease episode compared to non-inflammatory neurological diseases. Especially, CRP is used as a remission and therapy monitoring marker (55). Pathohistologically, the acute form is represented by a multifocal to generalized fibrinoid-necrotizing vasculitis with thrombosis and purulent leptomeningitis preferentially in the meninges of the cervical spinal cord (40, 43, 56–58).

The atypical, chronic, and protracted form of SRMA is observed primarily due to relapses and inadequate, immunosuppressive treatment. CSF analysis predominantly reveals mononuclear cells (44) and non-suppurative, mononuclear cell infiltrates in the meninges and perivascular tissue dominate pathohistological findings (58).

Steroid-responsive meningitis-arteritis offers ideal circumstances for the possible detection of ETs for the first time in canine central nervous system (CNS) tissue representing an immune-mediated, inflammatory, and non-infectious neuronal disorder mainly driven by a neutrophil immune response (40, 44). ET detection in the acute phase of Kawasaki disease of children, which causes a comparable vasculitis with consecutive inducing tissue damage-like SRMA, was successful (59–61). Consequently, we hypothesized that ETs take part in the etiopathogenesis of SRMA and a successful detection of ETs in the commonly affected tissues of meninges and vessels seems promising. This study should be a proof of principle that ETs can be detected in histologically confirmed cases of SRMA. Based on confirmation of extracellular DNA traps, corresponding clinical studies, new diagnostic, and treatment strategies could be developed.

## Materials and Methods

### Sample Collection

From the archive of the Department of Pathology, two dogs were selected for this study. The inclusion criteria were signalment, reported clinical signs, pathohistological findings such as purulent or lymphohistiocytic leptomeningitis with associated arteritis or periarteritis, and no detectable pathogens with special staining such as periodic acid–Schiff-reaction, Gram's staining, Ziehl–Neelsen's staining, or Grocott's silver impregnation method. Retrospective study revealed that no further microbiological or virologic examination was conducted on serum or CSF samples of these dogs to exclude a pathogenetic etiology.

### Histological Evaluation

Routinely processed formalin-fixed and paraffin-embedded (FFPE) tissues of the affected dogs were selected from the block archive of the Department of Pathology for further histopathologic and immunofluorescent examination.

Regions of affected tissue of the cervical spinal cord with associated leptomeningeal vessels and peripheral, particularly periadrenal vessels were embedded in paraffin and cut at 2–4 μm for H&E and immunofluorescence staining. H&E staining of the affected tissue was performed by automated dying in Leica ST4040 (Leica, Wetzlar, Germany) with 0.1% hematoxylin (Roth, Karlsruhe, Germany) and 1% eosin (Roth, Karlsruhe, Germany). The presence of vascular and meningeal lesions was evaluated qualitatively by a board certified veterinary pathologist [the European College of Veterinary Pathologists (ECVP)] with special emphasis on neutrophilic and inflammatory cell invasion in the vascular walls or meninges. The H&E slides were examined microscopically on an Olympus BX53 (Olympus, Tokyo, Japan) light microscope. Pictures were edited with ImageJ software (version 1.53, National Institutes of Health, USA).

### Extracellular Trap Examination

For ET detection, unstained, native paraffin slides of affected tissues of the spinal cord, brain, and periadrenal arteries were analyzed. Co-staining of DNA–histone-1 complexes and MPO or H3Cit was performed according to the following protocol as previously described (62, 63) with the following changes.

After permeabilization for 10 min (0.1% Triton X-100) and blocking for 20 min (blocking buffer for co-staining of DNA–histone-1 complexes and MPO: 5% bovine serum albumin, 5% goat serum, 2% cold water fish gelatin, 0.05% Tween-20, and 0.05% Triton X-100; blocking buffer for co-staining of DNA–histone-1 complexes and H3Cit: 10% fetal calf serum, 2% bovine serum albumin, 0.05% Tween-20, and 0.1% Triton X-100), samples were incubated overnight at 4°C using the following first antibodies, diluted in respective blocking buffer: mouse monoclonal IgG2a anti-DNA/histone (Millipore MAB3864, Billerica, Massachusetts, USA; 0.55 mg/ml; 1:100) and rabbit antihuman myeloperoxidase (Dako, A0398, 3.3 mg/ml, 1:300) or rabbit antihuman H3Cit (citrulline R2 + R8 + R17) antibody (Abcam, ab5103, Cambridge, UK, 1 mg/ml, 1:31.6). For isotype control, murine IgG2a (from murine myeloma M5409, Sigma Aldrich, Munich, Germany, 0.2 mg/ml, 1:36.4) and rabbit immunoglobulin G (IgG) (from rabbit serum I5006, Sigma Aldrich, Munich, Germany, 1.16 mg/ml, 1:108.75 for staining of DNA–histone-1 complex and MPO 1:36.7 for staining of DNA–histone-1 complex and H3Cit) were used. The secondary staining was performed for 1 h in the dark at room temperature using a goat anti-rabbit Alexa 633-conjugated antibody (Invitrogen, Carlsbad, California, USA, 2 mg/ml, 1:500) and a goat anti-mouse Alexa 488-conjugated antibody (Invitrogen, Carlsbad, California, USA, 2 mg/ml, 1:500). Counterstaining of DNA was performed with aqueous Hoechst 33342 (Sigma B-2261, St. Louis, Missouri, USA, 0.5 mg/ml, 1:1,000) for 10 min. At the end, all the samples were processed with the TrueVIEW Autofluorescence Quenching Kit (Vector laboratories, San Francisco, California, USA) following the manufacturer's instructions and covered with Mounting Medium of the TrueVIEW Autofluorescence Quenching Kit (Vector laboratories, San Francisco, California, USA).

Serial cuts of histopathologically altered tissues were stained and analyzed, whether ET formation or ET markers were detectable. Neutrophils and macrophages infiltrating the subarachnoid space, meningeal arteries, and extraneural perivascular tissue of SRMA-affected dogs are capable of releasing extracellular DNA fibers consisting of ET-markers such as DNA–histone-1 complexes, attached MPO, or H3Cit, which is a typical and strong evidence of ET formation (1, 64–66).

Extracellular trap formation was semiquantitatively analyzed in the meninges of the spinal cord and affected vessels. The amount of ET formation was counted in five 400 μm × 400 μm fields (0.16 μm^2^) of affected tissues of each dog and compared to each other (Table 1).

**Table 1:** Semiquantitative analysis of extracellular trap (ET) events in five representative immunofluorescent pictures.

		**Bernese mountain**		**Petit Basset Griffon**	
		**dog**		**Vendéen**	
**Localization**		**ET-events/**	**Average**	**ET-events/**	**Average**
		**0.16 μm^**2**^**		**0.16 μm^**2**^**	
Vessels	intraluminal	4, 1	2, 5	1, 1	1
	intramural	0, 0	0	0, 0	0
	perivascular	16, 4	10	11, 7	9, 5
Meninges		13, 10, 14	12, 3	25, 20, 22	22, 3

**Figure 1:**
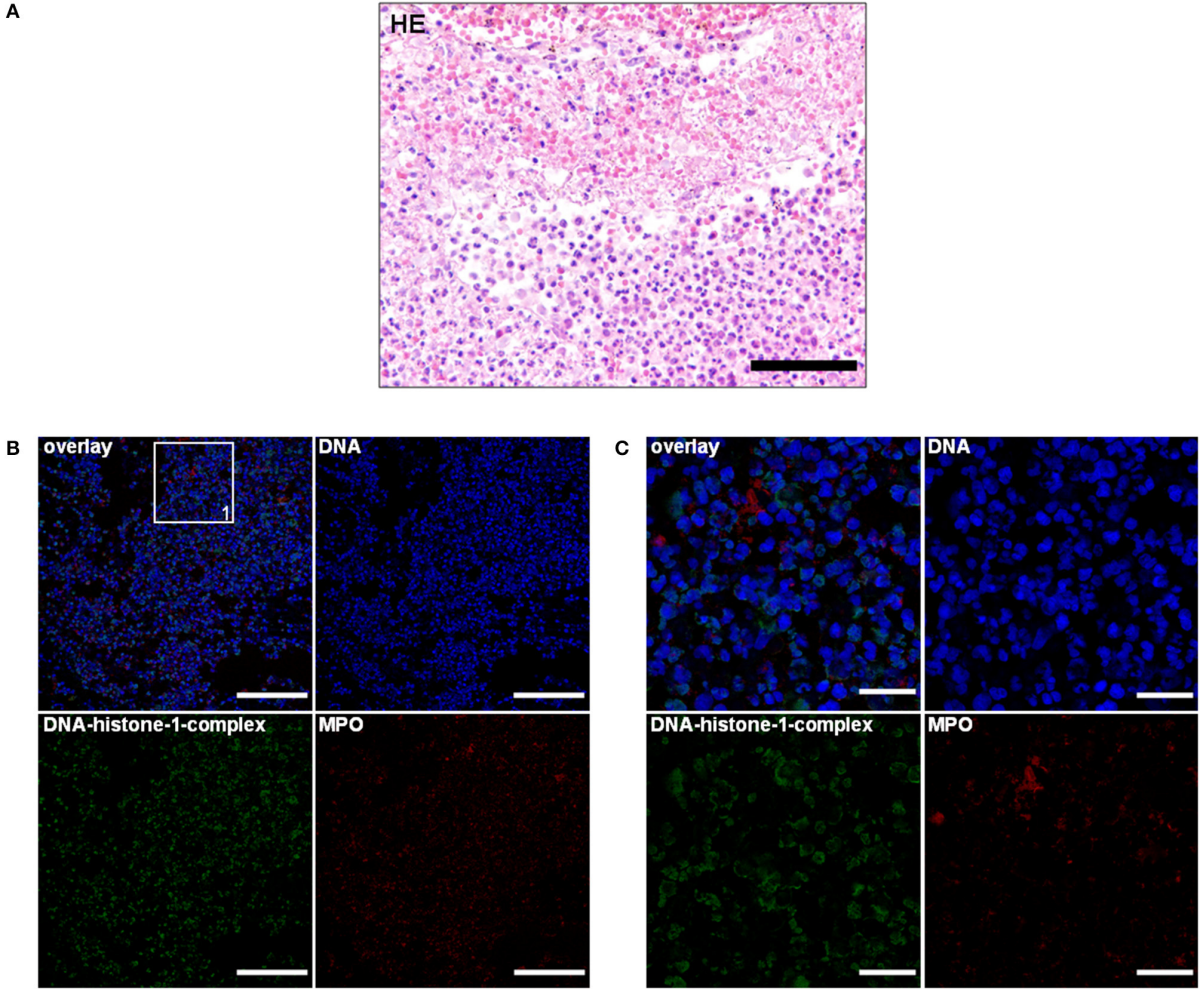
**(A)** An 11-month-old Bernese mountain dog. Histopathology. Cervical spinal cord: predominant infiltration of neutrophils and macrophages resulting in a moderate diffuse, purulent leptomeningitis. H&E. Scale bar = 100 μm. **(B)** Serial cuts of the concurrent formalin-fixed and paraffin-embedded (FFPE) block. Extracellular traps (ETs) as extracellular DNA fibers with associated myeloperoxidase (MPO) were present in the purulent leptomeningitis of the spinal cord. Settings of the immunofluorescence images were adjusted to a respective isotype control. Representative images are shown. IF. Blue, counterstaining of DNA (Hoechst), green, DNA–histone-1 complexes (ETs), and red, myeloperoxidase (MPO). Scale bar = 100 μm. **(C)** Zoom picture of area 1. IF. Scale bar = 20 μm.

*ET events were semiquantitatively analyzed by counting matching extracellular MPO or H3Cit and DNA-histone-1-complex singals in affected tissues in a square of 400 x 400 μm. Meninges and Vessels divided in intraluminal, intramural and perivascular events were seperately screened for ETs for each dog. Intraluminal there was no event detectable. Perivascularly and intraluminally there was no big difference in the counted ET-events. Meninges of the Petit Basset Griffon Vendéen were histopathologically (Figures 3A, 4A) and semiquantitatively more affected than the meninges of the Bernese mountain dog (Figure 1A)*.

### Immunofluorescence Microscopy

The stained samples were examined microscopically on a Leica TCS SP5 AOBS confocal inverted-base fluorescence microscope with HCX PL APO 40 × 0.75–1.25 oil immersion objectives with an Argon 405 and 633 nm laser (Leica, Wetzlar, Germany). The settings were adjusted using isotype control antibodies in separate preparations. Pictures were edited with ImageJ software (version 1.53, National Institutes of Health, USA).

## Results

### Signalment, History, Macroscopic, and Histopathological Findings

**Figure 2:**
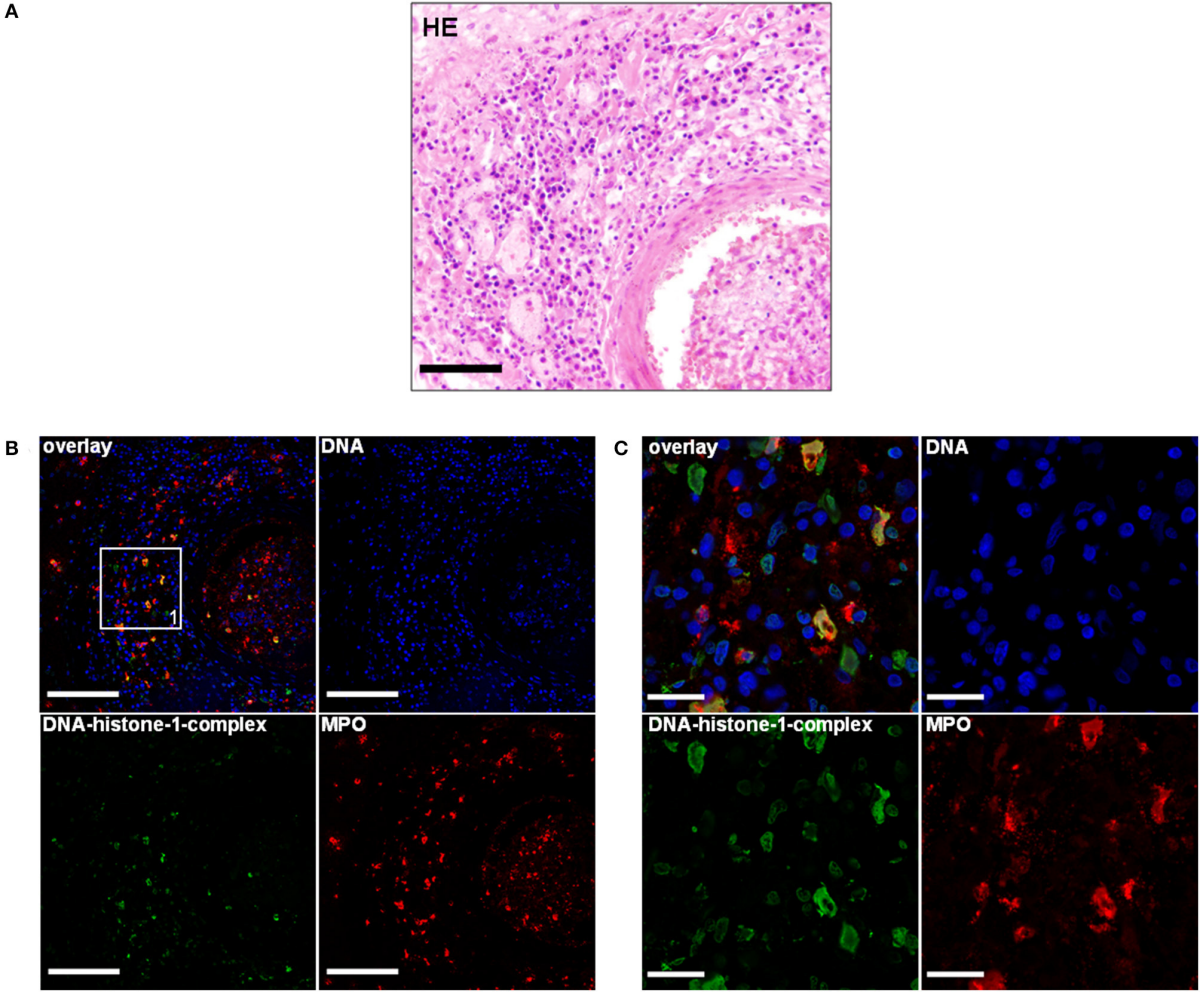
**(A)** An 11-month-old-Bernese mountain dog. Histopathology. Periadrenal artery: Moderate infiltration of lymphocytes, macrophages, and plasma cells causing a moderate, subacute to chronic, diffuse lymphohistiocytic, periadrenal periarteritis. H&E. Scale bar = 100 μm. **(B)** Serial of the concurrent FFPE block. Extracellular traps (ETs) as extracellular DNA fibers were detected in the perivascular region of a periadrenal artery. Settings of the immunofluorescence images were adjusted to a respective isotype control. Representative images are shown. Blue, counterstaining of DNA (Hoechst), green, DNA–histone-1 complexes (ETs), and red, myeloperoxidase (MPO). IF. Scale bar = 100 μm. **(C)** Zoom picture of area 1 is presented. IF. Scale bar = 20 μm.

Two dogs were included with histological lesions indicative of acute and chronic SRMA. The first dog was an 11 months old, male Bernese mountain dog. Anamnestically, this dog had recurrent episodes of pyrexia up to 41°C and lameness. Laboratory findings revealed moderate leukocytosis of 25,000/μl and elevated protein content of the CSF [positive Pandy-reaktion (+)]. Initial treatment was started with doxycycline and prednisolone for an unknown period and clinical signs were ameliorating. 3 weeks after terminating the medication, the dog relapsed and showed similar clinical signs with episodes of fever and leukocytosis of 28,000/μl. Another treatment with prednisolone was initiated and the dog was anesthetized for further diagnostics, but developed cardiorespiratory arrest. After unsuccessful resuscitation, it was sent to the Department of Pathology for necropsy. Necropsy revealed only agonal gross changes. Pathohistological evaluation of the cervical spinal cord revealed infiltration of neutrophils, macrophages, lymphocytes, and plasma cells resulting in a moderate diffuse, purulent, and lymphohistiocytic leptomeningitis (Figure 1A). Extraneural findings showed moderate infiltrations of lymphocytes, macrophages, and plasma cells causing a subacute to chronic, diffuse lymphohistiocytic, periadrenal periarteritis (Figure 2A).

**Figure 3:**
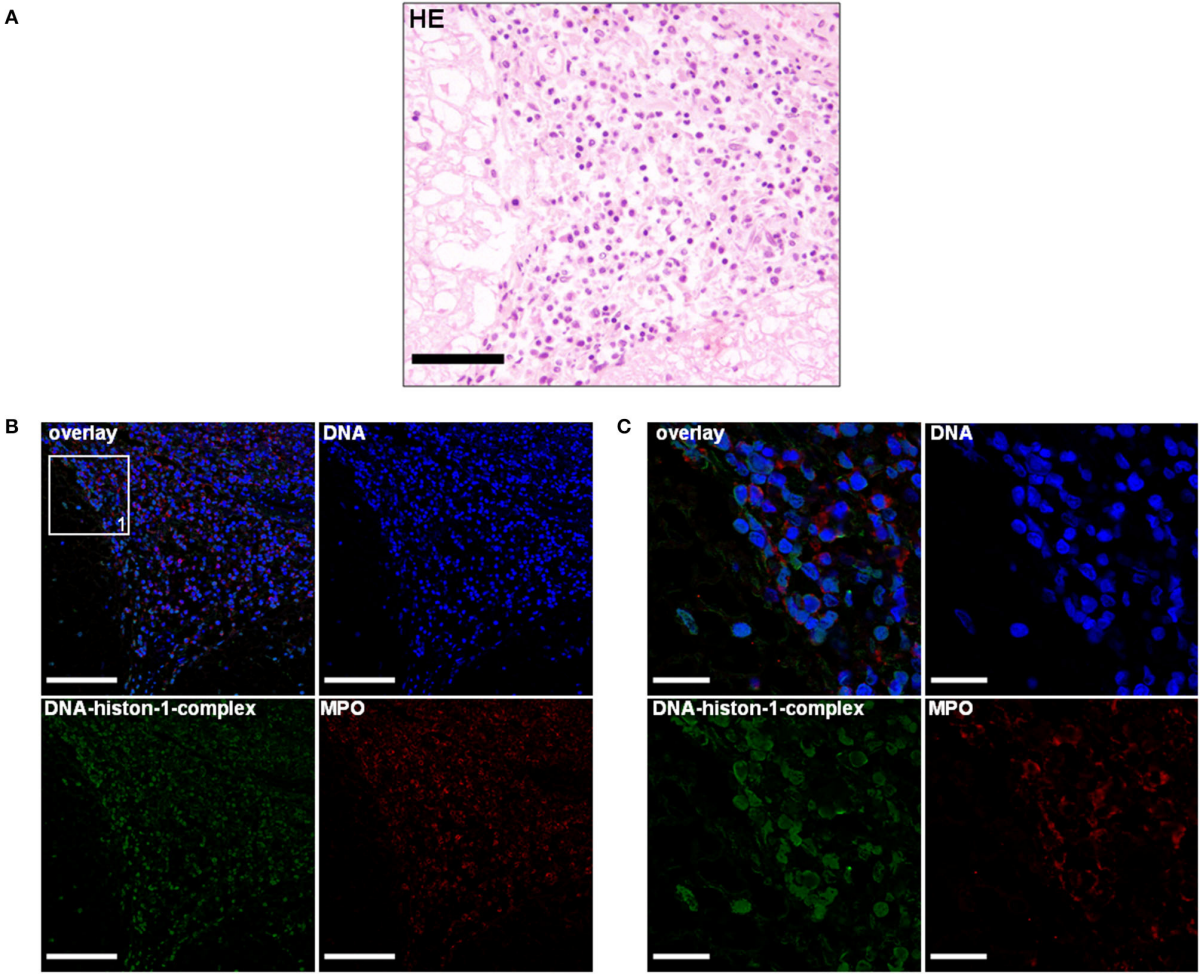
**(A)** A 5-month-old Petit Basset Griffon Vendéen. Histopathology. Cervical spinal cord: Moderate-to-severe infiltration of neutrophils, macrophages, lymphocytes, and plasma cells causing a severe, subacute, multifocal, lymphohistiocytic leptomeningitis H&E. Scale bar = 100 μm. **(B)** Serial cuts of the concurrent FFPE block. Extracellular traps (ETs) as extracellular DNA fibers and ETs markers were detected in the subarachnoid space. Settings of the immunofluorescence images were adjusted to a respective isotype control. Representative images are shown. IF. Blue, counterstaining of DNA (Hoechst), green, DNA–histone-1 complexes (ETs), and red, myeloperoxidase (MPO). Scale bar = 100 μm. **(C)** Zoom picture of area 1 is presented. Scale bar = 20 μm.

**Figure 4:**
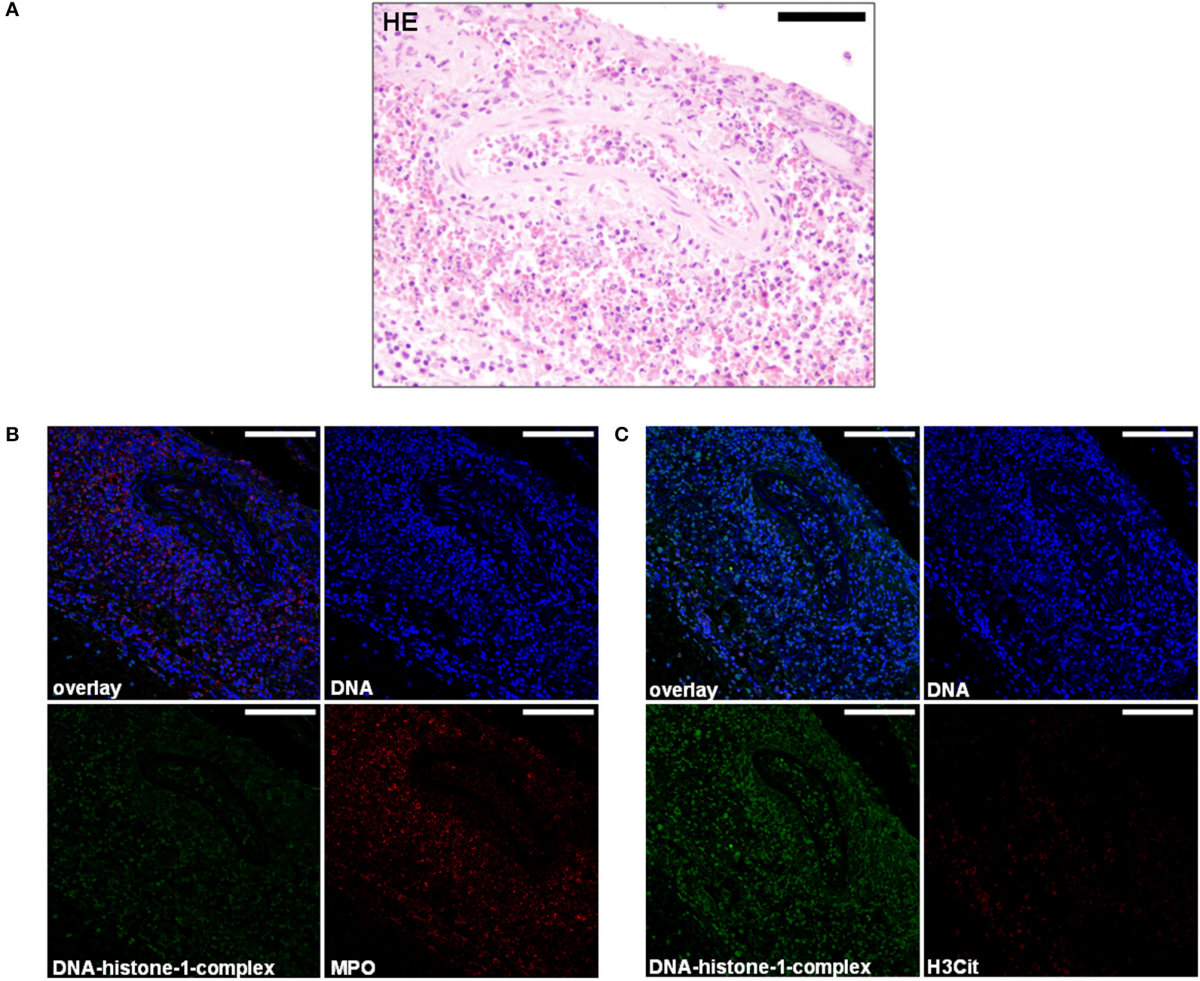
**(A)** A 5-month-old Petit Basset Griffon Vendéen. Histopathology. Cervical meningeal arteries revealed moderate perivascular infiltration of lymphocytes, macrophages, and a few neutrophils resulting in a moderate, acute, diffuse, purulent, lymphohistiocytic periarteritis. H&E. Scale bar = 100 μm. **(B)** Serial cuts of the concurrent FFPE block. Extracellular traps (ETs) as extracellular DNA fibers were detected in the subarachnoid space and perivasculary. Settings of the immunofluorescence images were adjusted to a respective isotype control. Representative images are shown. IF. Blue, counterstaining of DNA (Hoechst), green, DNA–histone-1 complexes (ETs), and red, myeloperoxidase (MPO). Scale bars = 100 μm. **(C)** Extracellular traps (ETs) as extracellular DNA fibers were detected in the subarachnoid space and perivasculary. Settings of the immunofluorescence images were adjusted to a respective isotype control. Representative images are shown. IF. Blue, counterstaining of DNA (Hoechst), green, DNA–histone-1 complexes (ETs), and red, citrullinated histone H3 (H3Cit). Scale bars = 100 μm.

The second dog was a 5-month-old, female, Petit Basset Griffon Vendéen, which was euthanized because of a pleural effusion causing dyspnea and additional acute kidney injury (urea in the aqueous humor: 180 mg/dl). The history revealed undulating fever episodes of unknown origin and relapsing episodes of forelimb lameness. No treatment strategies were attached to the submission report of this dog. Anamnestically, another littermate was affected with comparable clinical signs. Necropsy revealed diffuse subdural hemorrhage expanding from the cerebellum throughout the dural tube. A circumferential dark red mass was located in the precardiac mediastinum (4 cm × 4 cm × 3 cm). In the thoracic cavity, there was a hemothorax, consisting of partially clotted 300 ml in the left and 100 ml in the right pleural cavity. Pathohistologically the leptomeninx of the cervical spinal cord was moderately-to-severely infiltrated with neutrophils, macrophages, lymphocytes, and plasma cells showing a severe, subacute, multifocal, lymphohistiocytic leptomeningitis accompanied with severe subarachnoid hemorrhage with erythrophagocytosis (Figure 3A). Cervical meningeal arteries revealed mild periarterial infiltration of lymphocytes, macrophages, and few neutrophils resulting in a moderate, acute, diffuse, lymphohistiocytic periarteritis with an intraluminal thrombus formation and meningeal hemorrhage (Figure 4A).

Both the dogs showed features of chronic-active and acute neural and extraneural histopathologic lesions that are characteristically observed in undulating clinical courses of SRMA (44, 67). The severity of meningeal inflammatory infiltration of the Bernese mountain dog was lower than of the Petit Basset Griffon Vendéen.

### Immunofluorescence Findings in Meninges and Arteries of Canine Steroid-Responsive Meningitis-Arteritis

As a next step, we used fluorescence microscopy to visualize formation of ETs in the biopsies. Since the major backbone of ETs is DNA, DNA intercalating dyes are widely used to stain ETs based on the electrostatic interactions of these dyes, e.g., 4′, 6-diamidino-2-phenylindole (DAPI) with DNA (13). However, this method cannot discriminate between DNA derived from ET-releasing cells vs. necrosis. Furthermore, it has to be considered that some granule components such as antimicrobial peptides block the visualization of ETs by DNA-intercalating dyes (68). Therefore, antibody-based techniques that stain ET-specific markers such as DNA–histone complexes in combination with cell-specific proteins that are frequently found associated with ETs such as myeloperoxidase are needed to confirm release of ETs by immunofluorescence microscopy (13, 68–70).

Using this technique, the lymphohistiocytic, periadrenal periarteritis of the Bernese mountain dog showed mild presence of DNA–histone-1 complex positive web-like structures and moderate, extracellular MPO signal surrounding infiltrating macrophages. ET formation appeared perivascularly and intraluminally, but was not to be found in the vascular walls (Figures 2B,C; Table 1). These ET detections were similar to histopathological findings, which showed only perivascular inflammatory cell infiltration. Meningeal lesions occurred in contrast to perivascular lesions with mild infiltration of neutrophils next to macrophages and lymphocytes. Mild-to-moderate detection of extracellular DNA–histone-1 complexes and MPO as ET markers could visualized in the meninges of the spinal cord around infiltrating neutrophils (Figures 1B,C). To sum up pathohistological as well as immunofluorescent findings, ET formation could present in the meninges and extraneural arteries of this dog.

Extracellular trap markers in terms of extracellular DNA–histone-1 complexes and MPO were also positive in the meninges of the Petit Basset Griffon Vendéen. Infiltrating neutrophils, macrophages, and lymphocytes causing a purulent to lymphohistiocytic leptomeningitis are forming ETs proven by DNA–histone-1 complex, MPO, and H3Cit signals in this affected area (Figures 3B,C, 4B,C). Summarizing pathohistological and immunofluorescent findings of this dog, ETs were present at the time of death in damaged meninges and meningeal arteries. Respective isotype control images did not show any signal intensity at DNA–histone-1 complex, MPO, or H3Cit settings.

## Discussion

In this study, we could proof our hypothesis that ET formation and ET markers of two representative dogs suffering from SRMA are detectable and visualizable in typical affected tissues such as meninges of the cervical spinal cord and neural, as well as extraneural vessels. To the best of the author's knowledge, this is the first study confirming the presence of extracellular DNA formations composed of DNA–histone-1 complexes, MPO, or H3Cit in the CNS of dogs and especially affected with SRMA. ET formation was present in acute and chronic-active lesions of recurrent, waxing-waning disease periods of both the dogs, implicating that this mechanism of the immune system seems to play a certain role in the pathogenesis of SRMA.

As hallmark of the pathogenesis of SRMA, neutrophils conquer the subarachnoid space causing a neutrophil pleocytosis of the CSF (44). The detailed mechanism of this immune compartmentalization is not fully understood. Neutrophil recruitment to vascular wall adhesion is mediated by CD11a upregulation (51) and a possible factor of the blood–brain barrier disruption is caused by releasing matrix metalloproteinases-2 and−9 (MMP-2/-9) (71). Khandpur et al. (27) positively correlate the amount of netting neutrophils and production of interleukin-17 (IL-17). These findings could be supported by Freundt-Revilla et al. (72) that production of IL-17 ensures neutrophil granulocyte recruitment in the CNS compartment and disruption of the blood–brain barrier in dogs with SRMA. IL-17 production in dogs with SRMA can lead to increased NET formation and may facilitate the leukocyte extravasation of neutrophils by disrupting the blood–brain barrier.

We hypothesize that ET formation interdigitates with the current detailed knowledge of immunologic dysregulation causing SRMA (43). Recently, it was shown that ET formation promotes vasculopathies (73) and externalization of ET-associated proteins such as histones and MPO leads to vascular barrier injury (23, 74, 75). Especially, histones are described in small-vascular angiopathies to drive vascular damage and vascular wall necrosis (23, 74, 75). The positive evidence of H3Cit in the meningeal arteries could be another explanation to the invasion and compartmentalization of neutrophils in the subarachnoid space with associated hemorrhage and frequently detected fibrinoid-necrotizing arteritis in SRMA (43).

Furthermore, histones as major proinflammatory components of extracellular released DNA traps may drive and perpetuate the innate immune response and maintain persistent sterile inflammation in SRMA through interaction with Toll-like receptor (TLR) 4 (76–78). Being part of pattern recognition receptors (PRRs), TLRs play a crucial role of the innate immune system and stimulating the adaptive immune response (79). They are able to recognize foreign, pathogen-associated molecular patterns (PAMPs) in infectious diseases, as well as host-derived damage-associated molecular patterns (DAMPs) produced by tissue damage or cell death (80). Maiolini et al. (81) described higher expression of TLR-4 and TLR-9 on polymorphonuclear cells and monocytes in the acute stage of SRMA. The upregulation of this PRR on immune cells mediating the pathogenesis of SRMA such as neutrophils and macrophages illuminates chronic inflammation and autoimmunity and may be attributed due to higher levels of H3Cit (82, 83).

In addition, higher concentration of intrathecal produced extracellular heat shock protein 70 (eHSP70) as representative example of the DAMP family may interact with TLR4 (84). Continuous activation of neutrophils releasing their ETs due to interaction with DAMPs such as citrullinated histones or other host-derived self-antigens such as eHSP70 can lead to a vicious circle of autoimmunity and supporting the theory of existing autoantigenic triggers (14, 85). Based on these findings, the hypothesis of an existing self-antigen or environmental trigger acting according to the hit-and-run principle must be requested.

Furthermore, the externalization of ETs is the source of major autoantigens for autoantibody formation and is supposed to be pathogenic in several autoimmune-derived diseases (86). Until now, autoantibodies against endogenous CNS tissue only serve as “epiphenomenon” of SRMA (40, 87). If the presence of these major autoantigenetic in terms of ET-associated structures drive and maintain immunologic processes in meninges and vessels of these dogs, the complex pathogenesis of SRMA could be well explained.

Histopathological and immunofluorescent findings (Figures 1–4) of acute and chronic lesions in both the dogs represented by mainly lymphohistiocytic, as well as neutrophils invading meninges or perivascular spaces could be explained with prolonged activation of macrophages and neutrophils generating ETs or an impaired self-clearance of ETs. Remnants of ETs could serve as constant trigger in terms of DAMPs for immune cells maintaining CNS and vascular inflammation resulting in continuous invasion of neutrophils in this already chronic process. Both the dogs showed similar pathohistologic lesions of chronic active inflammation at the time of death with different amount of ET formation (Table 1). In general, the meninges of the Petit Basset Griffon Vendéen were infiltrated more severely than in the Bernese mountain dog. This could be explained by a more acute and severe clinical course of the Petit Basset Griffon Vendéen in contrast to the Bernese mountain dog. Another explanation could be the different pharmacological influence of variably administered anti-inflammatory drugs. Primarily perivascular detection of ETs was present in affected arteries.

Generating histological samples in the future will be very unlikely because clinical diagnosis, treatment management, and awareness of this disease reduced the mortality of SRMA in the last decades (44, 46, 88). Therefore, prospective clinical studies could confirm antemortem evidence of ETs in dogs measuring ET markers and correlating ET inducers in clinical accessible samples of serum and CSF such as H3Cit and cell-free DNA. Visualization or stimulation assays of ETs released by isolated nucleated cells in CSF samples in acute diseased, treated, and relapsed dogs with SRMA offer another possibility supporting the results of this study. Comparing ET markers to dogs with other inflammatory as well as non-inflammatory CNS diseases of infectious and non-infectious origin is necessary to probably underline and distinguish the final role of ETs in the pathogenesis of SRMA.

Treatment of autoimmune and immune-mediated diseases in veterinary medicine is lacking of specific therapeutic options such as the usage of recombinant monoclonal antibodies, intracellular pathway modulating, or receptor-targeting drugs (89). Also, steroid therapy is associated with many undesirable side effects and 30% of human patients are identified as “non-responders” or resistant to glucocorticoid application (88, 89). Specific ET-targeting therapeutics options with fewer side effects such as DNases (90) exist in human medicine and urge to be tested in veterinary medicine. SRMA represents an ideal large animal model of suppurative, non-infectious meningitis with proven ET formation to evaluate and develop new therapeutics in future research studies in a translational context (81). Based on this pilot study of ET formation in the CNS of dogs, clinical studies will be performed investigating the influence on canine neuropathies.

In conclusion, ETs are detectable in tissue samples of necropsied SRMA cases. This study represents the first trial to proof of principle of ET visualization in canine central nervous system tissue. The detection of ETs in SRMA gains new possibilities to explore the existence and etiopathogenetic influence of this host mechanism of immune cells in infectious and non-infectious canine neuropathies. To give an outlook, a magnitude of study is required concerning clinical importance of innovative diagnostics tools as remission and therapy marker and the development of specific, ET-targeting therapeutic options with fewer side effects than conventional glucocorticoid therapy.

## Data Availability Statement

The original contributions presented in the study are included in the article/Supplementary Material, further inquiries can be directed to the corresponding author.

## Author Contributions

AT, WB, MvK-B, and JN conceived, designed, and supervised the study. JW and MM performed all the experiments, analyzed the data, and performed immunofluorescence staining and microscopy. JW drafted the manuscript. PW performed histopathological examination. All authors contributed to the revision of the manuscript and have read and approved the final version of the manuscript.

## Funding

This Open Access publication was funded by the Deutsche Forschungsgemeinschaft (DFG, German Research Foundation) within the programme LE 824/10-1 “Open Access Publication Costs” and University of Veterinary Medicine Hannover, Foundation.

## Conflict of Interest

The authors declare that the research was conducted in the absence of any commercial or financial relationships that could be construed as a potential conflict of interest.

## Publisher's Note

All claims expressed in this article are solely those of the authors and do not necessarily represent those of their affiliated organizations, or those of the publisher, the editors and the reviewers. Any product that may be evaluated in this article, or claim that may be made by its manufacturer, is not guaranteed or endorsed by the publisher.
