# Supplementary material for: Detection of Extracellular Traps in Canine Steroid-Responsive Meningitis-Arteritis
Source: Front Vet Sci. 2022 May 3;9:863579. doi: 10.3389/fvets.2022.863579 (PMC9111528; doi:10.3389/fvets.2022.863579)
Supplement: Supplementary file 1 [file Data_Sheet_1.PDF]

Supplementary Material

Detection of extracellular traps in canine steroid-responsive meningitis-arteritis

Jan C. Wohlsein[1]*, Marita Meurer[2,3], Jasmin Neßler[1], Peter Wohlsein[4], Maren von-Köckritz-Blickwede[2,3], Wolfgang Baumgärtner[4] and Andrea Tipold[1]

[1]Department of Small Animal Medicine and Surgery, University of Veterinary Medicine Hannover, Foundation, 30559 Hannover, Germany

[2]Department of Biochemistry, University of Veterinary Medicine Hannover, Foundation, 30559 Hannover, Germany

[3]Research Center for Emerging Infections and Zoonoses, University of Veterinary Medicine Hannover, Foundation, 30559 Hannover, Germany

[4]Department of Pathology, University of Veterinary Medicine Hannover, Foundation, 30559 Hannover, Germany

*** Correspondence:**
Jan Christian Wohlsein
jan.christian.wohlsein@tiho-hannover.de

Supplementary Figures

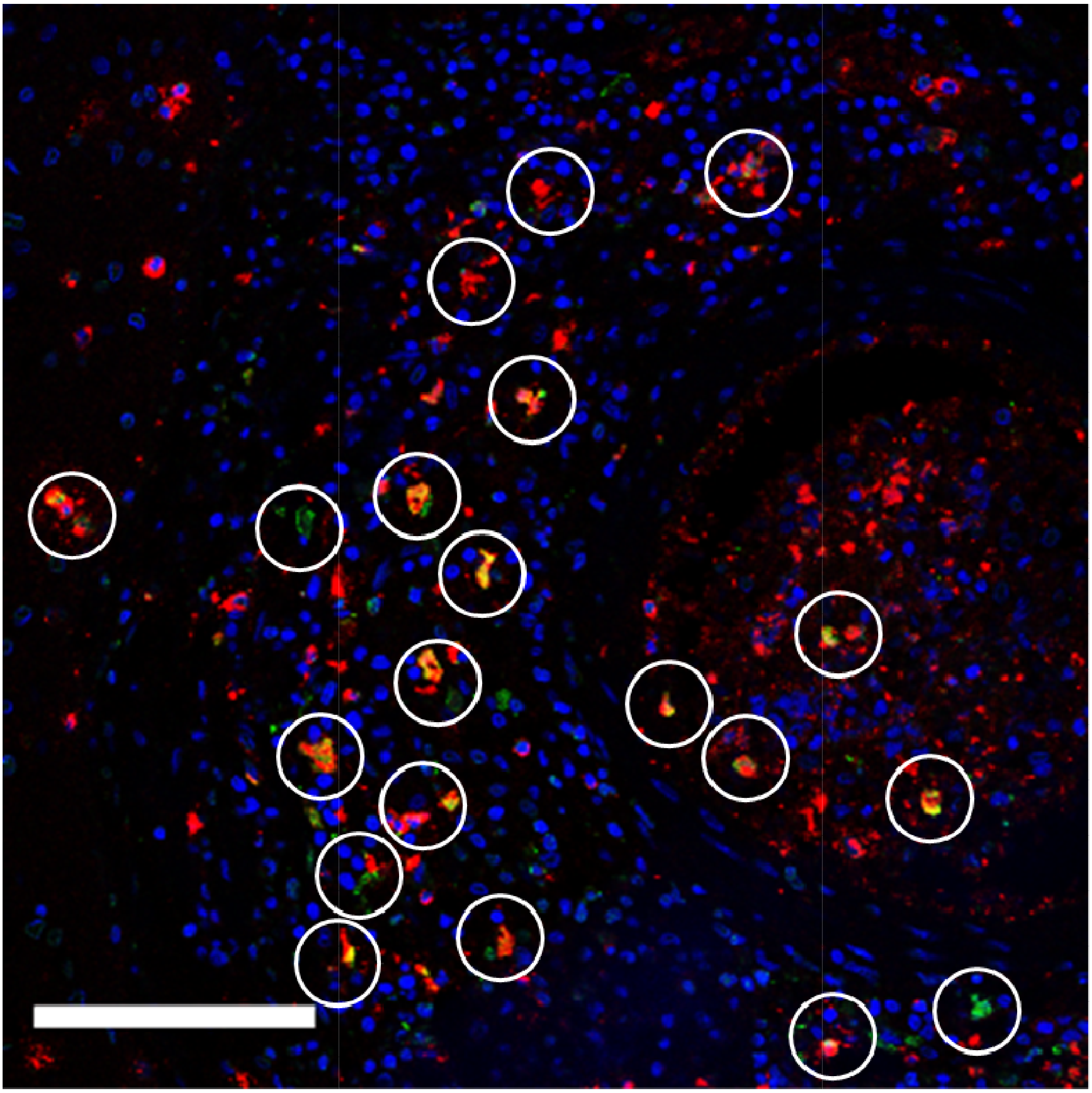

Supplementary Figure 1. Exemplary semiquantitative analysis of ET-events in five representative immunofluorescent pictures. Five representative images for each dog of 0,16 µm^2 were analyzed. White circle = ET-event. IF. Blue = counterstaining of DNA (Hoechst), green = DNA/histone-1-complexes (ETs), red = myeloperoxidase (MPO). Scale bar = 100 µm.
